# Application of autofluorescence robotic histology for quantitative evaluation of the 3‐dimensional morphology of murine articular cartilage

**DOI:** 10.1002/jemt.22948

**Published:** 2017-09-30

**Authors:** Patricia Das Neves Borges, Tonia L. Vincent, Massimo Marenzana

**Affiliations:** ^1^ Department of Bioengineering Imperial College London London United Kingdom; ^2^ Kennedy Institute of Rheumatology, Nuffield Department of Orthopaedics, Rheumatology and Musculoskeletal Sciences University of Oxford, Roosevelt Drive Oxford United Kingdom; ^3^ Present address: Present address School of Health and Social Care ‐ Allied Health Sciences London South Bank University, 103 Borough Road London, SE1 0AA United Kingdom

**Keywords:** automated image analysis, destabilization of medial meniscus model, fluorescence imaging, high‐throughput 3D histomorphometry, osteoarthritis

## Abstract

Murine models of osteoarthritis (OA) are increasingly important for understating pathogenesis and for testing new therapeutic approaches. Their translational potential is, however, limited by the reduced size of mouse limbs which requires a much higher resolution to evaluate their articular cartilage compared to clinical imaging tools. In experimental models, this tissue has been predominantly assessed by time‐consuming histopathology using standardized semi‐quantitative scoring systems. This study aimed to develop a novel imaging method for 3‐dimensional (3D) histology of mouse articular cartilage, using a robotic system—termed here “3D histocutter”—which automatically sections tissue samples and serially acquires fluorescence microscopy images of each section. Tibiae dissected from C57Bl/6 mice, either naïve or OA‐induced by surgical destabilization of the medial meniscus (DMM), were imaged using the 3D histocutter by exploiting tissue autofluorescence. Accuracy of 3D imaging was validated by *ex vivo* contrast‐enhanced micro‐CT and sensitivity to lesion detection compared with conventional histology. Reconstructions of tibiae obtained from 3D histocutter serial sections showed an excellent agreement with contrast‐enhanced micro‐CT reconstructions. Furthermore, osteoarthritic features, including articular cartilage loss and osteophytes, were also visualized. An in‐house developed software allowed to automatically evaluate articular cartilage morphology, eliminating the subjectivity associated to semi‐quantitative scoring and considerably increasing analysis throughput. The novelty of this methodology is, not only the increased throughput in imaging and evaluating mouse articular cartilage morphology starting from conventionally embedded samples, but also the ability to add the third dimension to conventional histomorphometry which might be useful to improve disease assessment in the model.

## 1 INTRODUCTION

Osteoarthritis (OA) is a worldwide leading cause of joint impairment and pain that primarily involves articular cartilage degradation, subchondral bone changes, and osteophyte formation (Goldring, 2012). Despite its high prevalence, sensitive biomarkers of early disease are missing (Lotz et al., 2013) and most cases are identified once moderate radiographic evidence of articular cartilage loss and bone remodeling are established (Roth, Wirth, Emmanuel, Culvenor, & Eckstein, 2017). Semi‐quantitative grading of radiographic images is often used to evaluate disease progression (Felson et al., 2005; Roemer, Eckstein, Hayashi, & Guermazi, 2014), but, additionally to the lack of sensitivity to track temporal changes (Guermazi, Hayashi, Roemer, & Felson, 2013), scoring fails to provide a direct measure of articular cartilage loss (Brem et al., 2009). Magnetic resonance imaging (MRI) due to its 3D capability of visualizing not only articular cartilage loss, but also meniscal extrusion and bone marrow lesions (Blumenkrantz & Majumdar, 2007) is increasingly applied for semi‐quantitative (Guermazi et al., 2015) and quantitative (Favre et al., 2017; Raynauld et al., 2008) assessment of OA. Volumetric measurements of articular cartilage can be obtained upon segmentation (Brisson et al., 2017; Favre et al., 2017) and these have demonstrated sensitivity to detect disease from its early stages (Amin et al., 2005), emphasizing the importance of 3D quantitation in disease assessment.

Preclinical models play a key role in OA studies, not only to study disease progression from its onset, in which clinical data is often scarce, but also to test new therapeutic approaches (Fang & Beier, 2014; Gregory et al., 2012; Vincent et al., 2012). As the mouse is amenable to genetic manipulation, it is widely used as an experimental model, nevertheless its translational potential is limited by its small size (Fang & Beier, 2014). Clinical imaging tools, namely MRI, cannot be used to evaluate articular cartilage due to their insufficient resolution. Conventionally, the degradation of articular cartilage is assessed by time‐consuming histopathology and lesions graded depending on depth and extent of surface involvement using standardized semi‐quantitative scoring systems (Li et al., 2016; Poulet, Westerhof, Hamilton, Shefelbine, & Pitsillides, 2013). While informative, histological scoring across the joints has inter‐ and intra‐observer variability (Pritzker et al., 2006) and morphological measurements obtained from 2D sections, such as articular cartilage area (McNulty et al., 2011) and thickness (Ko et al., 2013; Poulet et al., 2013), may be influenced by tissue orientation during processing (David et al., 2017). Furthermore, manual segmentation of regions‐of‐interest is often required. Therefore, methodologies that image mouse articular cartilage in 3D, with high‐throughput and with quantifiable measurements are desirable. Most of these methodologies have relied on the combination of 3D X‐ray imaging, such as micro‐computed tomography (micro‐CT) or phase‐contrast, with contrast enhancement agents to improve the visualization of the tissue of interest (Lusic & Grinstaff, 2013). Use of enhancement agents is required because, being radiolucent, the articular cartilage is undetectable by conventional X‐ray sources. A number of different contrast agents have been used with success in *ex vivo* joint imaging by micro‐CT (Das Neves Borges, Forte, Vincent, Dini, & Marenzana, 2014; Kerckhofs, Sainz, Wevers, Van de Putte, & Schrooten, 2013; Kotwal, Li, Sandy, Plaas, & Sumner, 2012; Lakin et al., 2016) and phase‐contrast (Ruan et al., 2013). Additionally, monochromatic, high flux and parallel radiation beams used in synchrotron phase‐contrast imaging have demonstrated the ability to image mouse articular cartilage in the absence of any contrast agent (Li et al., 2014; Marenzana et al., 2014). Alternative modalities to X‐ray imaging include optical projection tomography combined with fluorescent labeling of the articular cartilage extracellular matrix (Inagawa et al., 2009) and autofluorescence imaging by confocal laser scanning microscopy (Stok et al., 2009). Measures such as articular cartilage thickness, volume, and surface area have been obtained by these 3D techniques and, potentially, these can achieve higher sensitivity than scoring in the detection of changes, similar to the observations in clinical studies.

In this work, we report the development of a novel method for quantitative evaluation of the 3D morphology of mouse articular cartilage. We used a robotic imaging system with high‐resolution capability, termed “3D histocutter” (Department of Bioengineering, Imperial College London), that automatically sections embedded tissue samples and serially acquires fluorescence microscopy images (Burgoyne, Downs, Bellezza, & Hart, 2004). Preliminary studies have demonstrated its ability to image decalcified mouse knee joints (Murienne et al., 2011) and, subsequently, its applicability on soft tissue imaging, namely in brain (Tang, Ardila Jimenez, Chakraborty, & Schultz, 2016) and lung (Andrikakou, Vickraman, & Arora, 2016) samples from experimental models. Furthermore, analogous automated systems have successfully imaged mouse embryos (Tsuchiya & Yamada, 2014) and rat trabecular bone (Matheny et al., 2013; Slyfield, Tkachenko, Wilson, & Hernandez, 2012) in 3D using fluorescence. Therefore, we sought to extend this emergent imaging principle to the articular cartilage of undecalcified mouse knee joints. We exploited tissue autofluorescence to produce images using the microscope included in the 3D histocutter and applied this high‐throughput imaging method to a surgical mouse model of knee OA to determine its usefulness in visualizing articular cartilage lesions. To obtain quantitative data on tissue morphology, we developed a bespoke image analysis software. Finally, we validated our findings against conventional histopathology and *ex vivo* contrast‐enhanced micro‐CT (PTA‐CT) imaging (Das Neves Borges et al., 2014).

## 2 MATERIALS AND METHODS

### 2.1 Surgical destabilization of the medial meniscus and sample preparation

Experimental work was conducted in the UK according to the Animals (Scientific Procedures) Act (1986) and subjected to Home Office approval. Male C57Bl/6 mice (10‐week old, *n* = 8) underwent surgical destabilization of medial meniscus (DMM) on the right knee joint (Glasson, Blanchet, & Morris, 2007), while the left limb was used as a control (contralateral). At 20‐weeks postsurgery, animals were sacrificed, knee joints harvested, tibiae dissected and fixed in 70% ethanol. Samples were subsequently divided in two groups: while the first group (*n* = 4) was used for 3D robotic histology (undecalcified), the second group (*n* = 4) was used for conventional histology (decalcified). For robotic histology, tissue preparation involved 24 hr of dehydration in 100% ethanol followed by cold infiltration in methyl methacrylate (Chappard, Palle, Alexandre, Vico, & Riffat, 1987). Modifications to the original protocol included the addition of 1% Sudan Black B to opacify the blocks and reduce background autofluorescence during image acquisition. Histological blocks were kept at 4°C and polymerization required approximately 3 weeks. For conventional histology, tibiae were decalcified in 10% ethylenediaminetetraacetic acid, dehydrated, and embedded in paraffin. Coronal sections were taken at regular spacing across the samples (80 µm between levels in a total of twelve levels) and stained using Safranin‐O. A group of naïve male C57Bl/6 mice (*n* = 4) was sacrificed at 10‐weeks of age, tibiae dissected and fixed to be used for direct comparison between 3D robotic histology and *ex vivo* contrast‐enhanced micro‐CT using PTA (Das Neves Borges et al., 2014). The same procedure of opaque embedding was performed upon contrast‐enhanced micro‐CT.

## 3 3D ROBOTIC HISTOLOGY

### 3.1 Experimental set‐up

**Figure 1:**
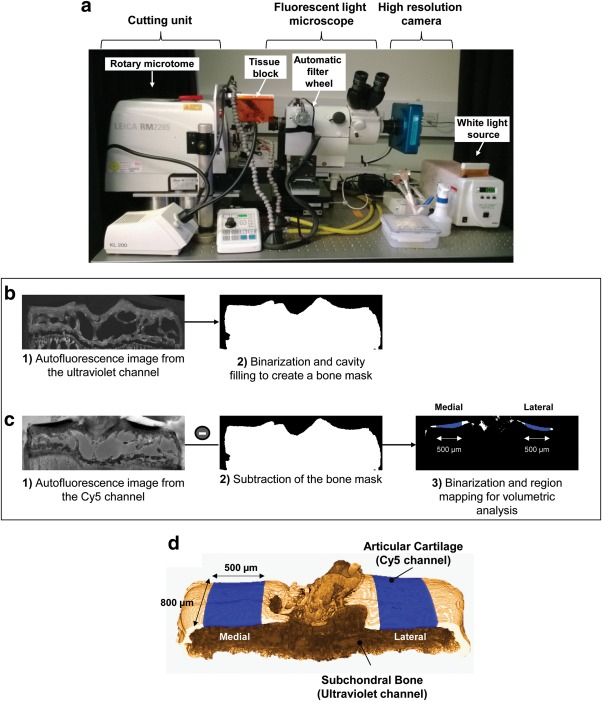
(a) 3D histocutter set‐up. The rotary microtome in the cutting unit is coupled to a fluorescent light microscope and a high‐resolution camera that consecutively images the histological block surface. The motor in the filter wheel automatically switches between fluorescent channels—ultraviolet, fluorescein isothiocyanate (FITC), Texas Red and near‐infrared cyanine (Cy5), and the whole system is controlled by bespoke Java software running in a standard computer. (b) Flowchart of the image processing method used to segment and quantify articular cartilage using autofluorescence in the 3D histocutter. Upon re‐scaling in XY‐axis, images from the ultraviolet channel were used to create a “bone mask” to segment articular cartilage from the Cy5 channel images. After image subtraction, two volumes‐of‐interest were mapped for volumetric quantification (c). (d) 3D model of a mouse tibial epiphysis obtained by dual autofluorescence in the 3D histocutter. Subchondral bone, imaged by the ultraviolet channel and articular cartilage, imaged by the Cy5 channel, were overlaid and the automated volumes‐of‐interest (500 µm in width by 800 µm in length) in the center of the medial and lateral aspects of the tibial plateau are color‐coded in blue

The 3D histocutter (Department of Bioengineering, Imperial College London) set‐up is based on previous designs (Burgoyne et al., 2004; Weninger & Mohun, 2002) and composed of a cutting unit coupled to a fluorescent light microscope with a white light source (arc lamp) and a high‐resolution camera (Figure 1a). The motor in the filter wheel automatically switches between the four fluorescent filters available—ultraviolet, fluorescein isothiocyanate (FITC), Texas Red and near‐infrared cyanine (Cy5)—and the system is controlled by a standard computer running in‐house Java software. The 3D histocutter uses a “cut'n view” approach to automatically section the histological block of embedded tissue and image the surface that is continuously maintained in focus. While the rotary microtome in the cutting unit removes thin sections that are discarded by air puffers, the high‐resolution camera images the surface. A sequential stack of images is recorded in the computer and acquisition using multiple fluorescent channels is also possible (Andrikakou et al., 2016; Murienne et al., 2011; Tang et al., 2016).

### 3.2 Image acquisition

The microtome was set with a cutting thickness of 5 µm and the total depth of cut along the *Z*‐axis was 2,000 µm (400 slices). Two fluorescent channels, the ultraviolet and Cy5, were used to image subchondral bone and articular cartilage, respectively. The choice of these two channels was based on our preliminary tests that demonstrated the high autofluorescence levels of subchondral bone when excited by ultraviolet light (Kazakia et al., 2007; van Gaalen et al., 2010), while near‐infrared fluorescence from the Cy5 demonstrated very low autofluorescence of the articular cartilage compared with subchondral bone and background. Hence, both tissues could be clearly distinguished and for segmentation purposes, this channel was chosen for articular cartilage imaging. Exposure time in each channel was selected upon analysis of the histogram of the gray level intensity to avoid saturation of the signal; while in the ultraviolet channel exposure time was in 0.8 seconds, in the Cy5 10 seconds of exposure were required. The fluorescent light microscope was set with a 3× optical zoom and 2× magnifying lens, achieving a XY resolution of 1.218 µm/pixel. Sectioning and imaging run automatically and unattended, and approximately 6 hours per sample were required to obtain images from the two fluorescent channels.

### 3.3 Image processing

Prior volumetric analysis, datasets from both channels were rescaled in the XY‐axis to achieve an isotropic voxel size of 5 µm^3^. Images from the ultraviolet channel (Figure 1b) were used to create a mask to segment subchondral bone from the images obtained from the Cy5 channel. Gaussian filtering, followed by threshold (Otsu, 1975), morphological operations, and cavity filling were applied to create a binary mask that enclosed all bone tissue. This mask was subtracted from the Cy5 dataset (Figure 1c) and following additional image processing procedures (Gaussian filtering, inversion, and threshold), the articular cartilage layer was automatically segmented. For volumetric quantification, two fixed‐size volumes‐of‐interest (500 µm in width across 800 µm in the anterior‐posterior axis, Figure 1d) were mapped in the center of the medial and lateral aspects of the tibial plateau (Das Neves Borges et al., 2014) and the average thickness calculated using ImageJ (National Institutes of Health, Bethesda, Maryland, USA) (Doube et al., 2010).

### 3.4 PTA‐CT: Image acquisition and quantification

The protocol used for articular cartilage imaging by PTA‐CT has been described in detail elsewhere (Das Neves Borges et al., 2014). Briefly, tibiae were incubated in PTA at room temperature for 24 hr and scanned in a micro‐CT scanner (SkyScan1172 X‐ray microtomograph, Antwerp, Belgium) within PTA (5 µm/pixel, 50 kV, 200 µA). Reconstructed scans were analyzed to quantify the average thickness of the articular cartilage in the load‐bearing regions using the automated methodology previously reported (Das Neves Borges et al., 2014).

### 3.5 Histological scoring

Images of the medial and lateral aspects of the plateau in DMM‐operated and contralateral tibiae were acquired by light microscopy (10× magnification) and graded depending on the degree of cartilage damage according to a scoring system previously detailed (Chia et al., 2009; Inglis et al., 2008) which was modified from the semi‐quantitative system originally defined by Glasson et al. (Glasson et al., 2005). The final score for each aspect of the plateau was obtained from the sum of the scores of all levels.

### 3.6 Statistical analysis

Statistics were computed using GraphPad Prism 7.02 software (San Diego, CA, USA) and data expressed as mean ± standard error of the mean (*SEM*). Differences between naïve, 20‐weeks post‐DMM and contralateral tibiae were determined using one‐way analysis of variance (ANOVA) followed by *post hoc* Tukey's multiple comparisons tests. Two‐tails, paired Student's *t*‐tests were applied in the comparisons between PTA‐CT and 3D histology measurements of articular cartilage thickness in naïve animals. Since histopathology scores did not follow normal distributions, which was tested by Shapiro‐Wilk tests, non‐parametric analysis by Friedman's tests with Dunn's post‐tests was applied to test differences between scores. Values were considered statistically different at *p < .05, **p < .01, ***p < .001, and ****p < .0001.

## 4 RESULTS

### 4.1 3D robotic histology

**Figure 2:**
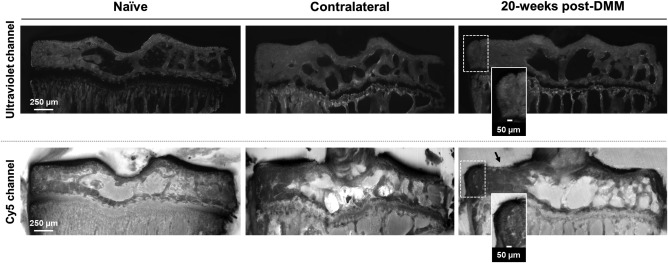
3D imaging of mouse tibiae using dual autofluorescence in the 3D histocutter. Representative coronal views of a naïve, contralateral and 20‐weeks post‐DMM tibia obtained in the ultraviolet and Cy5 fluorescent channels for subchondral bone and articular cartilage imaging, respectively. The black arrow in the Cy5 channel of DMM‐operated indicates the presence of a lesion in the medial articular cartilage, while the white dashed boxes indicate the presence of a marginal medial osteophyte, visible in both fluorescent channels and highlighted in the zoomed views for further detail

3D robotic histology demonstrated the ability to correctly visualize tissue morphology in mouse tibiae. The ultraviolet channel imaged subchondral bone microstructure in detail (upper row of images in Figure 2), whereas articular cartilage was visible in the Cy5 channel as the structure presenting the lowest fluorescent signal (lower row of images in Figure 2). Articular cartilage cellularity was not visible in any of the two fluorescent channels. OA lesions, including articular cartilage loss (black arrow in the Cy5 channel, Figure 2) and marginal osteophytes (dashed white boxes in both channels and zoomed views for further detail, Figure 2) were identified in DMM tibiae and confined to the medial aspect of the plateau. At such late time point of the pathology, experimental samples showed full erosion of the hyaline articular cartilage down to the calcified cartilage zone in the load‐bearing regions, while the contralateral remained intact. Naïve and contralateral tibiae presented similar morphology and the lateral side remained spared in all samples.

### 4.2 3D measurements of the articular cartilage

**Figure 3:**
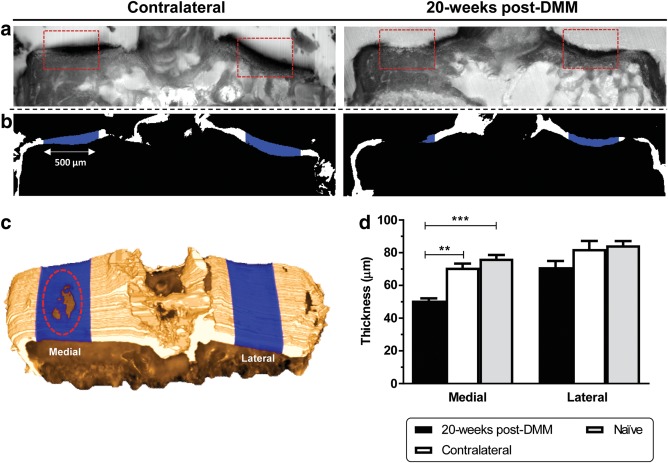
Automated analysis of articular cartilage in the load‐bearing regions using dual autofluorescence in the 3D histocutter. (a) Representative coronal views of a pair of contralateral/DMM tibiae imaged by 3D robotic histology in the Cy5 channel and (b) automated mappings (500 μm in width) in the load‐bearing areas for volumetric analysis. Dashed boxes in (a) indicate the areas of interest where articular cartilage was assessed. (c) Top view of a 3D model of the mouse tibia at 20‐weeks post‐DMM showing the mappings in the center of the tibial plateau. Note the full erosion of the articular cartilage caused by OA in the medial side, exposing the subchondral bone plate (highlighted by the dashed ellipse). (d) Column bar graph of the mean articular cartilage thickness measured in contralateral, DMM and naïve samples (*SEM* error bars, *n* = 4; **p < .01 and ***p < .001, obtained by one‐way ANOVA followed by *post hoc* Tukey's multiple comparisons tests)

High‐throughput segmentation and mapping of articular cartilage (Figure 3a) was achieved by combining image processing procedures in the two fluorescence channels (Figure 3b). Thickness measurements within the automated volumes‐of‐interest showed significant loss of medial articular cartilage in DMM‐operated when compared with contralateral (*p* = .0012 for DMM vs. contralateral, Figure 3d). Severe erosion caused this thinning down to the tidemark level and changes were confined to the load‐bearing area (enhanced by the dashed ellipse in the 3D model, Figure 3c), thus confirming the qualitative observations. Additionally, thickness measurements in contralateral and naïve animals were similar (*p* = .74 for medial contralateral vs. naïve, and *p* = .99 for lateral contralateral vs. naïve, Figure 3d).

### 4.3 Validation by contrast‐enhanced micro‐CT using phosphotungstic acid

**Figure 4:**
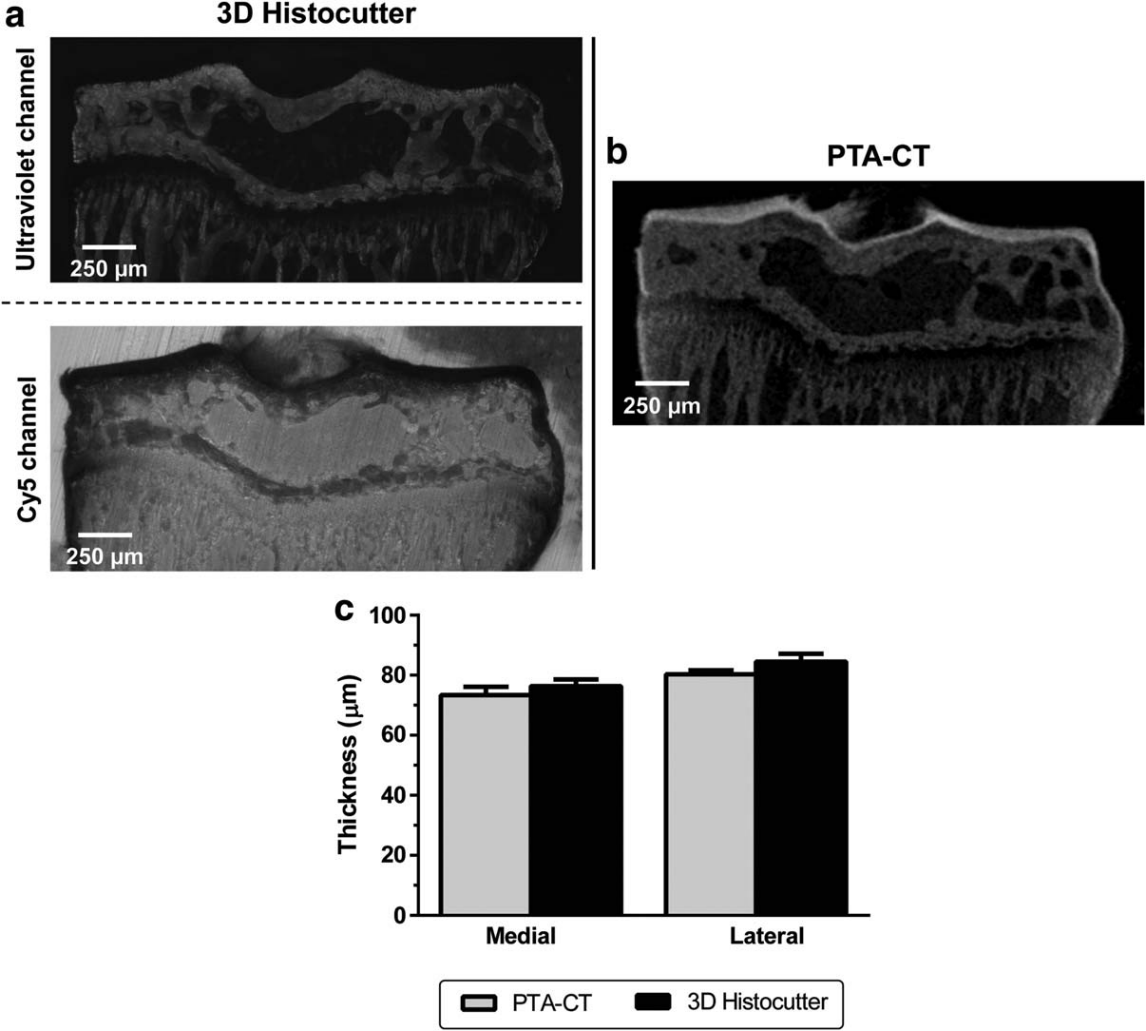
Articular cartilage imaging by 3D robotic histology and PTA‐CT. Representative coronal views of a mouse tibia imaged by (a) dual autofluorescence (ultraviolet and Cy5 channels) in the 3D histocutter upon imaging by (b) PTA‐CT (50 kV, 200 µA, 5µm/pixel). (c) Column bar graph of the mean articular cartilage thickness measured using the two imaging modalities and applying similar quantitative methods based on mapping of volumes‐of‐interest (*SEM* error bars, *n* = 4; *p* < .05 by two tails, paired, Student's *t*‐tests)

To validate the measurements obtained by 3D robotic histology, we compared the quantification using PTA‐CT and 3D histology across similar volumes‐of‐interest. Very good qualitative agreement was observed between the two imaging modalities (Figure 4a,b), where we identified similar features in articular cartilage and subchondral bone structure in both techniques. Measurements of articular cartilage thickness using the two modalities were also in good agreement (*p* = .45 for medial PTA‐CT *vs*. 3D histocutter, and *p* = .10 for lateral PTA‐CT vs. 3D histocutter, Figure 4c).

### 4.4 Histopathology

**Figure 5:**
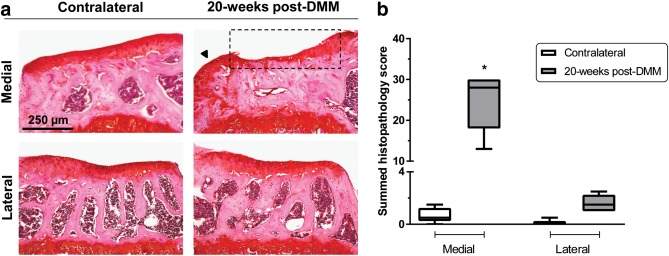
(a) Representative coronal views of the medial and lateral aspects of the tibial plateau in 20‐weeks post‐DMM and contralateral tibiae (10× magnification, Safranin‐O staining), showing lesions in the medial articular cartilage of DMM‐operated, which is enhanced by the dashed box. The black arrowhead indicates the presence of a medial osteophyte in the DMM‐operated. (b) Boxplot of the summed histopathology scores in the medial and lateral aspects of the tibial plateau of DMM‐operated and contralateral tibiae. Symbols represent the means and *SEM* error bars (*n* = 5, *p < .05 by Friedman's tests and Dunn's post‐tests for differences between medial/lateral contralateral vs. DMM)

Histopathology showed lesions in medial articular cartilage of DMM‐operated tibiae (Figure 5a), but not on the lateral side, similar to the results obtained using the 3D histocutter (Figure 2). At 20‐weeks post‐DMM, marked articular cartilage loss down to the tidemark was visible, leading to significantly elevated scores when compared with the corresponding contralateral (Figure 5b), and thus confirming disease induction.

## 5 DISCUSSION

In this study, we sought to image mouse articular cartilage in 3D and obtain morphological information on this tissue using a customized device for high‐throughput robotic histology. The use of automated systems for high‐resolution, 3D histological imaging is becoming increasingly important to assess experimental models of pathology (Tsuchiya & Yamada, 2014) and newer systems can now achieve full automation on serial sectioning and imaging (Kazakia et al., 2007; Murienne et al., 2011; Wilson et al., 2008) which previous systems did only by semi‐automated approaches (Hashimoto, Kusakabe, & Ishikawa, 2008). Using the 3D histocutter, we successfully demonstrated that, in the absence of any staining and purely based on tissue autofluorescence, articular cartilage and subchondral bone can be imaged in 3D at high‐resolution. Furthermore, osteoarthritic features, namely articular cartilage degradation and osteophyte formation, were also clearly visualized using this customized imaging system. Our observations on bone imaging using ultraviolet light are consistent with the work of Slyfield et al. in a rat model (Slyfield et al., 2012), while articular cartilage reconstructions are in line with the findings reported by Stok et al. (Stok et al., 2009) using confocal microscopy. Automated segmentation and quantitation of the articular cartilage was possible through the combination of image processing procedures in the two fluorescence channels used for image acquisition and mapping of volumes‐of‐interest, similar to the approach used for PTA‐CT (Das Neves Borges et al., 2014). Images in the ultraviolet channel were acquired with the specific purpose of creating accurate masks for bone subtraction in the Cy5 channel, thereby assuring that the structure segmented was confined to the articular cartilage and did not include other structures. While confocal microscopy imaging has also demonstrated the ability to quantify mouse articular cartilage based on tissue autofluorescence (Moodie, Stok, Muller, Vincent, & Shefelbine, 2011; Stok et al., 2009), this requires stitching procedures to reconstruct the entire volume of the tibial plateau. In addition, the articular cartilage must be manually segmented. Confocal microscopy has further limitations on the thickness imaged as well as on the axial resolution achieved (Hashimoto et al., 2008; Zhang et al., 2016). These limitations are completely overcome using the 3D histocutter system, and sequential whole‐structure sectioning/imaging can be concatenated with automated segmentation and 3D reconstruction. In the group of surgically‐induced OA, thickness measurements were significantly reduced in the medial articular cartilage of operated compared with contralateral and naïve joints. Articular cartilage erosion was enhanced in the 3D models where areas of full thickness loss down to the subchondral bone plate were visible in the medial load‐bearing areas. Furthermore, we confirmed disease induction by conventional histology and similar lesions were observed using both histological methods. Qualitative observations and quantitative data from 3D histology were also in good agreement with PTA‐CT imaging, enabling further validation of the technique.

Limitations of the current study must, however, be considered. Since this imaging modality is based on a histological technique, it is destructive with sections being discarded in the process, and involves procedures of tissue preparation, namely dehydration and embedding. Furthermore, only one late time point upon OA induction was studied and therefore, we cannot determine the sensitivity of the method to early articular cartilage lesions. Additionally, despite clearly showing articular cartilage morphology, the channels used for image acquisition did not provide information on tissue cellularity, such as chondrocyte morphology and number, which are also altered in OA (Goldring & Goldring, 2010; McNulty et al., 2011). Future developments of the method could include the staining of the samples prior to embedding, for example, with nuclear staining to assess cellularity (Steyer, Roy, Salvado, Stone, & Wilson, 2009; Zhang et al., 2016) or with matrix‐specific fluorescent labels to assess protein distribution in 3D. Additionally, this methodology could be extended to the whole joint and femoral articular cartilage degradation can also be evaluated. Finally, although we did not explore the quantitation of subchondral bone, it has been shown that 3D histomorphometry using autofluorescence can assess formation and resorption events on cancellous bone (Slyfield et al., 2012; Tkachenko et al., 2009). Therefore, the 3D histocutter could be useful to further investigate osteoarthritic alterations on subchondral bone, which are known to occur from the early stages of disease (Das Neves Borges, Vincent, & Marenzana, 2017).

In conclusion, we have demonstrated that customized histomorphometry can be used to image mouse articular cartilage in 3D purely based on tissue autofluorescence. The novelty of this method is not only the increased throughput in sectioning and imaging applied to mouse articular cartilage, but also in the ability of screening lesions in 3D. Furthermore, the contrast between articular cartilage and underlying subchondral bone achieved by this technique enables accurate segmentation and fast‐throughput analysis, overcoming the major limitations of conventional histopathology assessment of articular cartilage in the mouse model.

## CONFLICT OF INTEREST

The authors have no conflict of interest to declare.
